# Efficacy and Safety of First-Line Chemotherapies for Patients With Advanced Biliary Tract Carcinoma: A Systematic Review and Network Meta-Analysis

**DOI:** 10.3389/fonc.2021.736113

**Published:** 2021-09-28

**Authors:** Yanfeng Jiang, Zhiming Zeng, Jie Zeng, Cuizhen Liu, Jinfeng Qiu, Ye Li, Jing Tang, Ning Mo, Lihua Du, Jie Ma

**Affiliations:** ^1^Department of Oncology, First Affiliated Hospital of Guangxi Medical University, Nanning, China; ^2^Department of Oncology, Liuzhou People’s Hospital, Liuzhou, China

**Keywords:** biliary tract carcinoma, chemotherapies, first-line, network meta-analysis, adverse events

## Abstract

**Background:**

At present, chemotherapy is still the primary treatment for advanced biliary tract carcinoma, but it is challenging to balance the efficacy and side effects. Network meta-analysis (NMA) is a better way to identify the protocol, and the advantage is that it can be combined with direct and indirect evidence to judge the best treatment regimens. Therefore, we conducted NMA on the searched randomized controlled trials (RCTs).

**Methods:**

NMA was conducted regarding the searched RCTs by comparing progression-free survival (PFS), overall survival (OS), objective remission rates (ORRs), and adverse events (AEs) of different chemotherapy protocols.

**Results:**

We screened 24 studies that met the inclusion criteria for further analysis. Compared with other regimens, the best supportive care (BSC) or FUFA protocol has a worse OS. Folfox4, GEMOX+erlotinib, and C+GEMOX can improve patients’ PFS compared with BSC. Patients receiving GP+cediranib protocol have higher ORRs. There was reduced neutropenia grade ≥3 when adopting GP+cediranib, GS, C+GEMOX, RAM+GP, and MER+GP than when using FUFA protocol. The probability of vomiting of XELOX is lower than that of GEM+XELOX. There is a lower diarrhea incidence of XELOX than that of GEMOX+erlotinib. The results of cluster grade analysis illustrated that GEMOX+erlotinib owned a higher ORR and a higher surface under the cumulative ranking (SUCRA) of neutropenia and vomiting but also had a lower SUCRA of diarrhea and fatigue. Meanwhile, both GEMOX and C+GEMOX have a better ORR and a higher AE SUCRA.

**Conclusion:**

The NMA demonstrated that chemotherapy combined with targeted therapy has better efficacy and lower incidence of AEs than chemotherapy alone.

## Introduction

Biliary tract carcinoma (BTC) can be divided into intrahepatic cholangiocarcinoma (ICC), extrahepatic cholangiocarcinoma (ECC), gallbladder carcinoma, and ampullary cancer according to the position of disease occurrence, in which ECC can be further divided into hepatic portal cholangiocarcinoma and distal cholangiocarcinoma. BTC is a malignant tumor originating in the bile duct epithelium, accounting for 3% of all digestive tract tumors (1, 2). The global incidence of BTC is on the rise, especially in Asian countries. Because of the hidden disease and atypical early clinical symptoms, the patients are primarily in the middle and terminal stage of the disease when diagnosed, and the overall prognosis is poor, with a 5-year survival rate lower than 5%. A diagnosis of advanced BTC means that it is challenging to conduct surgical resection. Therefore, palliative chemotherapy is a more vital treatment to improve the survival rate and patients’ life quality.

Currently, based on the results of randomized controlled ABC-02 and JCOG1113/FUGA-BT in period III, gemcitabine combined with cisplatin or gemcitabine combined with tegafur, gimeracil, oteracil potassium capsules is recommended for the first-line treatment of advanced BTC (3, 4). Based on some results of period II findings, other optional two-drug-combined first-line therapeutic protocols include the combination of gemcitabine with oxaliplatin, oxaliplatin with 5-FU, gemcitabine with capecitabine, oxaliplatin with capecitabine, and so on (5–7).

Targeted drugs and immune checkpoint inhibitors (ICIs) have been explored in BTC treatment, and there is no evidence of adjuvant therapy. In terms of the first-line treatment of advanced BTC, the combination of ICIs with chemotherapy or targeted drugs is still in the clinical trial stage. Hence, at present, chemotherapy is still the primary treatment for advanced BTC, but it is challenging to balance the efficacy and side effects, and the evidence-based medical evidence for drugs used in advanced BTC therapy is still less. The efficacy and safety of many chemotherapy regimens are still controversial. Additionally, the evidence-based medical evidence for medications used in advanced BTC therapy is still inadequate.

Network meta-analysis (NMA) is a better way to identify the protocol. The advantage is that it can be combined with direct and indirect evidence to judge the best treatment plan instead of relying solely on direct head-to-head comparisons of the two drugs. Therefore, we conducted NMA on the searched randomized controlled trials (RCTs). Progression-free survival (PFS), overall survival (OS), objective remission rates (ORRs), and adverse events (AEs) of different chemotherapy protocols were compared to find the best treatment strategy for the first-line treatment of advanced BTC in these clinical studies (8).

## Materials And Methods

### Search Strategy

We have retrieved all articles published before August 10, 2020, in PubMed, EMBASE, and Cochrane Library. The combination of subject words and free words was adopted for retrieval with the following MeSH terms: “Bile Duct Neoplasms”, “Cholangiocarcinoma”, “Gallbladder Neoplasms,” “advanced”, “unresectable”, “randomized”.

### Selection Criteria

The literature should meet the following criteria: (1) study type: published RCT of II/III period; (2) subjects: advanced BTC confirmed by histology; (3) patients with advanced BTC receiving first-line chemotherapy; (4) the primary outcome indicators: reported PFS, OS, and ORR; the secondary outcome indicators: the probability of neutropenia level ≥3, vomiting, diarrhea, and fatigue. The exclusion criteria are as follows: (1) letters, reviews, case reports, non-human studies, and articles that do not provide raw data; (2) non-English articles; (3) non-randomized controlled single-arm studies; (4) research includes a comparison of chemotherapy with adjuvant or neoadjuvant treatment; (5) the study only included patients with specific gene mutations.

### Data Extraction and Quality Assessment

Data were extracted independently by two researchers, and the following information was recorded in the Microsoft Excel spreadsheet: study number, first author, year of publication, country, pathological diagnosis, patient’s tumor stage and Eastern Cooperative Oncology Group (ECOG) score, patient’s sex, age, sample size of each group, sample size, treatment plan, usage of test group and control group, hazard ratio (HR) and 95% CI of PFS and/or OS, and occurrence of the probability of neutropenia grade ≥3, vomiting, diarrhea, and fatigue.

Two researchers independently assessed the quality of all included literature based on RCT Cochrane Reviewer bias risk assessment criteria: (1) generation of random sequences; (2) allocation concealment or not; (3) blind method or not; (4) complete results or not; (5) selective reporting or not; (6) other biases. These key points are divided into three levels: low risk, high risk, and unclear risk. Differences between investigators are resolved through discussion.

### Statistical Analysis

HR and its 95% CI were adopted to express outcome indicators of time–event variables (PFS and OS); OR and corresponding 95% CI were applied to represent the outcome indicators of binary variables (ORR and adverse reactions). Engauge4.1 was used to estimate the HR and its 95% CI if only survival curves were provided. Stata14.0 software was adopted to make an evidence relationship diagram of NMA, generating network diagram for different protocols. R software gemtc installation package and gemtc software were applied to conduct NMA based on Bayesian framework. Considering the heterogeneity between the studies, the random-effects consistency model was used for all data analyses. The parameters of R software are as follows: four simulated chains, 400,000 simulated iterations, 50,000 adjustment iterations, 10 refinement iterations. The 95% CI was adopted as a criterion judging whether the difference was statistically significant: When 95% CI was over 1, it prompts P < 0.05, the difference was not statistically significant, and *vice versa*.

We assessed heterogeneity and inconsistency between studies using the I^2^ statistic and p-value within a visual forest plot to time–event variables. p > 0.05 is considered to have no inconsistency. With I^2^ values below 25%, between 25% and 50%, and above 50%, the heterogeneity is deemed to be low, moderate, or high, respectively. For binary variables, we used node-splitting analysis to assess heterogeneity and inconsistency. We investigated the inconsistency factor (IF) among studies in each closed loop. If the 95% CIs of IF values include zero, it indicates no significant inconsistency. The τ^2^ = 0 and p > 0.05 are considered to have no inconsistency and low heterogeneity. A funnel plot evaluated the publication bias.

Within the Bayesian framework, the overall ranking of treatments was estimated through NMA by calculating the ranking probability of each method. We calculate the surface under the cumulative ranking (SUCRA) within the framework of frequency science to estimate the overall ranking of treatment. Cluster rank analysis was conducted on the ORR of each intervention and the corresponding reported SUCRA of AEs (neutropenia level ≥3, vomiting, diarrhea, and fatigue).

## Results

### Study Selection and Characteristics

**Figure 1:**
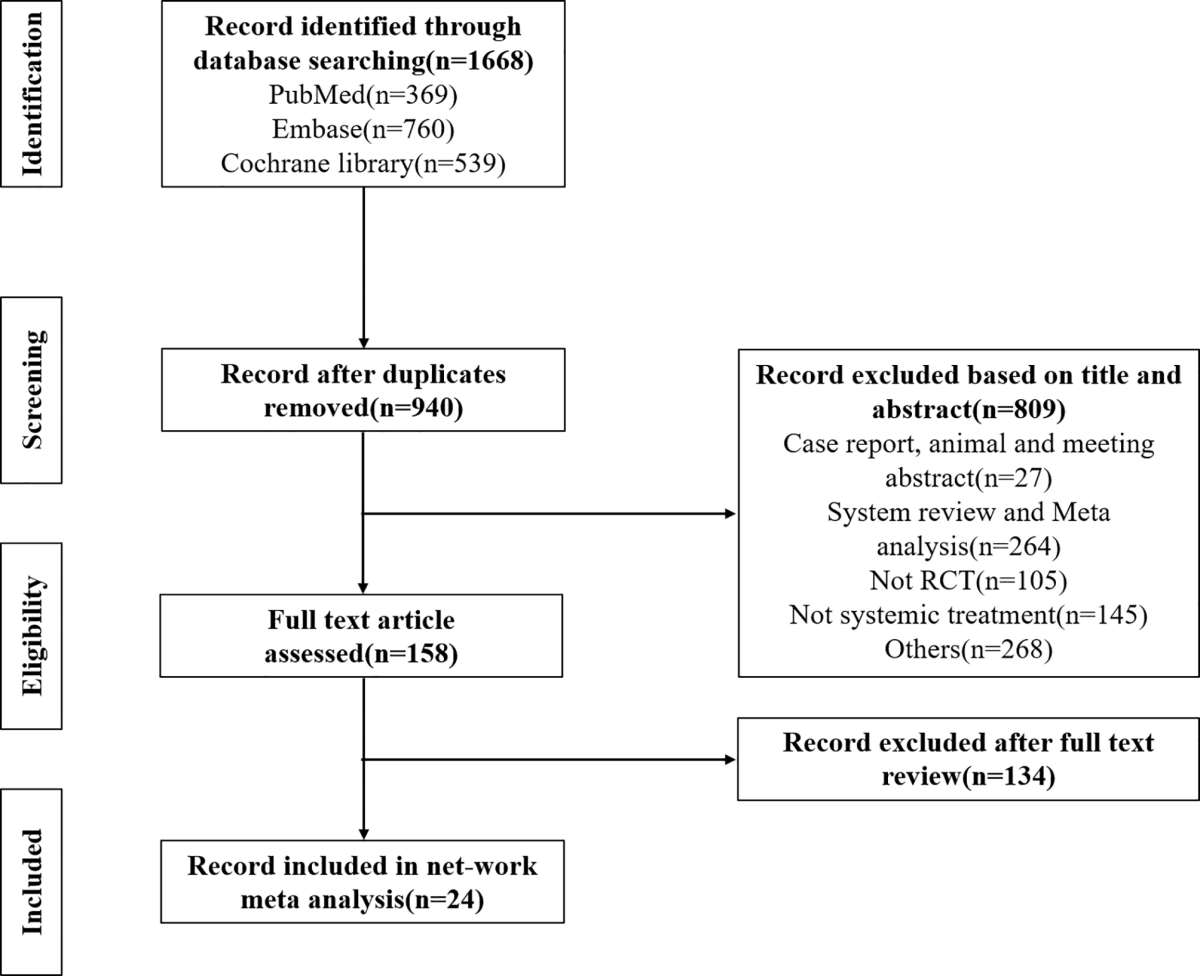
Literature search, screening process, and results chart.

We searched a total of 1,668 records in the database and removed 701 duplicate documents. After reading the title and abstract, we preliminarily screened 967 papers. The title/summary and the full text were screened based on inclusion and exclusion criteria. Finally, we screened 24 studies that met the inclusion criteria for further analysis. The flowchart of literature screening is shown in **Figure 1**. **Table 1** summarizes the basic features included in the study. The studies covered the period from 2010 to 2020, included 3,555 patients with advanced BTC, and involved 20 treatments, including best supportive care (BSC), FUFA, GEMOX, XELOX, GP+cediranib, GP, GEM, IP, GS, S1, Folfox4, C+GEMOX, GEMOX+erlotinib, FP+radiotherapy, GEM+sorafenib, SP, GPS, GEM+XELOX, RAM+GP, and MER+GP. The specific usage of each treatment has been described in **Table 1**. Bias risk for the overall study can be seen in **Supplementary Figures S1A, S1B**.

**Table 1:** Characteristic and treatment characteristic of the included studies.

Study(phase, country)	Regimens	Patients(N)	Male (N)	Median age(Y)	mPFS (M)	mOS (M)	ORR (%)	≥3grade Neutropenia (N)	Dosage
Sharma et al., (9)	FUFA	28	5	47	3.5	4.6	14.3	2	FUFA: 5-FU: 425mg/m²+LV 20mg/m² intravenous (IV) bolus weekly for 30 weeks
(India III)	Gemox	27	5	49	8.5	9.5	30.7	10	GEMOX: Gemcitabine: 900mg/m²+Oxaliplatin: 80 mg/m²
	BSC	27	6	51	2.8	4.5	0		
Kim et al., (5)	Gemox	114	70	64	5.3	10.4	24.6	16	GEMOX: Gemcitabine: 1000mg/m²/d1, d8+Oxaliplatin: 100 mg/m²/d1。
(Korea III)	Xelox	108	74	62	5.8	10.6	15.7	5	XELOX: Capecitabine: 1000mg/m²/d1-14 bid po+Oxaliplatin: 130mg/m²/d1
Valle et al., (10)	GP+cediranib	62	34	68	8	14.1	44	26	Cisplatin: 25 mg/m²+Gemcitabine: 1000 mg/m² d1, 8
(UK II)	GP	62	28	65	7.4	11.9	19	23	Cediranib: 20mg qd po
Valle et al., (3)	GP	204	96	64	8	11.7	26.1	50	GP: Cisplatin: 25mg/m^2^+Gemcitabine: 1000mg/m², d1, 8
(UK III)	GEM	206	98	63	5	8.1	15.5	33	GEM: Gemcitabine: 1000mg/m², d1, 8, 15
Dos Santos et al., (11)	IP	24			5.3	11.9	35		IP: CPT-11: 65mg/m² d1, 8+Cisplatin 60mg/m² d1
(Brazil II)	GP	23			7.8	9.8	31.8		GP: Gemcitabine: 1000mg/m² d1, 8+Cisplatin 25mg/m² d1
Huang et al., (8)	GS	32	19		5.6	8.2	18.8	21	GS: Gemcitabine: 1000 mg/m² d1, 15+S1: 80‐120mg/m², bid po d1-14
(China)	GP	34	22		6.5	10.2	20.6	20	GP: Gemcitabine: 1000 mg/m² d1, 8+Cisplatin 25mg/m², d1, 8 q3w
Morizane et al., (4)	GP	175	99	67	5.8	13.4	32.4	104	GS: Gemcitabine: 1000 mg/m², d1, 8+S1:80‐100mg/m², bid po d1-14
(Japan III)	GS	179	97	67	6.8	15.1	29.8	106	GP: Gemcitabine: 1000 mg/m², d1, 8+Cisplatin: 25mg/m², d1, 8
Li et al., (12)	GS	25	19	57	4.9	11	36		Gemcitabine: 1000 mg/m², d1, 8, 15
(China)	GEM	25	16	55	3.7	10	24		S1: 80‐100mg/m², bid po d1-14
	S1	25	19	57	1.6	6	8		
Schinzari et al., (6)	FUFA	23	10	61	2.8	7.5	21.7	1	LV: 100 mg/m² d1, 2, q2w
(Italy II)	Folfox 4	25	11	62	5.2	13	28	2	5-FU: 400 mg/m² d1, 2+5-FU: 1200 mg/m² (46 hours infusion)
	BSC	25	13	65			0		Oxaliplatin: 85 mg/m² d1, q2w
Okusaka et al., (13)	GP	41	18	65	5.8	11.2	19.5	17	GEM: Gemcitabine: 1000mg/m² d1,8,15
(Japan II)	GEM	42	21	66	3.7	7.7	11.9	11	GP: Gemcitabine: 1000mg/m² d1, 8+Cisplatin: 25mg/m² d1, 8
Malka et al., (14)	C-GEMOX	76	43	61	6.1	11	23	61	GEMOX: Gemcitabine: 1000 mg/m² d1+Oxaliplatin 100 mg/m² d1
(France II)	GEMOX	74	42	62	5.5	12.4	23	57	Cetuximab 500 mg/m² d1 or d2
Lee et al., (15)	GEMOX+erlotinib	135	91	59	5.8	9.5	30	3	GEMOX: Gemcitabine: 1000 mg/m² d1+Oxaliplatin 100 mg/m² d2
(South Korea III)	GEMOX	133	79	61	4.2	9.5	16	5	Erlotinib 100 mg qd
Philip et al., (16)	FP+radiotherapy	18	7	69	5.8	13.5		0	CHRT: radiotherapy: 50 Gy in 25 fractions, 5 days a week+5 FU, 300 mg/m²+Cisplatin 20 mg/m² d1-4 and d29-32 (Cisplatin 80 mg/m² at day 1 or 2 and day 29 or 30)
(France II)	GEMOX	16	8	75	11	19.9		4	GEMOX: Gemcitabine: 1000 mg/m² d1+Oxaliplatin: 100 mg/m² d1, q2w
Moehler et al., (17)	GEM+sorafenib	49	29	64	3	8.4	14	33	Gemcitabine 1000 mg/m²
(Germany II)	GEM	48	25	65	4.9	11.2	10	35	Sorafenib: 400mg, bid, po
Chen et al., (18)	C-GEMOX	62	28	61	6.7	10.6	27	11	GEMOX: Gemcitabine: 800 mg/m², d1+Oxaliplatin: 85mg/m², d1
(Taiwan II)	GEMOX	60	30	59	4.1	9.8	15	2	Cetuximab: 500mg/m², d1
Novarino et al., (19)	Folfox 4	22	11	62	5.4	14.1	13.6	6	Folfox4: Oxaliplatin: 85mg/m² d1+LV 200mg/m² d1, 2+5-FU: 400/600mg/m²/d1-2
(Italy)	GEM	18	12	65	3.9	8.3	0	5	Gemcitabine: 1250 mg/m²
Sasaki et al., (20)	GS	30	16	68	5.6	8.9	20	10	GS: Gemcitabine: 1000 mg/m², d1, 15+S1 80‐120mg/m², bid po d1-14 q4w
(Japan II)	GEM	32	20	75	4.3	9.2	9.4	7	GEM: Gemcitabine: 1000 mg/m², d1, 8, 15 q4w
Morizane et al., (21)	GS	51	27	66	7.1	12.5	36.4	31	GS: Gemcitabine: 1000 mg/m², d1, 8+S1: 60‐100mg/m², bid po d1-14
(Japan II)	S1	50	28	63	4.2	9	17.4	2	S1: 80-120mg/m², po bid for 4 weeks, followed by a 2-week rest
Morizane et al., (21)	GP	49	31	59	5.7	10.1	9	24	GP: Gemcitabine: 1000mg/m² d1, 8+Cisplatin: 60mg/m² d1
(Korea II)	SP	47	31	60	5.4	9.9	10	14	SP: S1 80‐120mg/m2, bid po d1-14+Cisplatin: 60mg/m² d1
Sakai et al., (22)	GPS				7.4	13.5	41.5		GPS: Gemcitabine: 1000mg/m² d1+Cisplatin: 25 mg/m²+S1: 80mg/m² d1-7
(Japan III)	GP				5.5	12.6	15		GC: Gemcitabine 1000 mg/m² d1, 8+Cisplatin 25 mg/m² d1, 8
Markussen et al., (23)	XELOX+GEM	47	23	65	5.7	8.7	17	1	XELOX+GEM: Oxaliplatin: 50 mg/m²+Gemcitabine: 1000 mg/m²+Capecitabine: 650 mg/m², bid, d1-14
(Denmark II)	GP	49	23	65	7.3	12	16	21	GP: Cisplatin: 25 mg/m², d1, 8+Gemcitabine: 1000mg/m² d1, 8
Kim et al., (24)	GEMOX	54	35	62	3	8	10		GEMOX: Gemcitabine: 1000 mg/m²+Oxaliplatin: 100mg/m²
(Korea III)	GEMOX+erlotinib	49	33	59	6.1	10.2	20		Erlotinib: 100 mg qd po
Valle et al., (25)	RAM+GP	106			6.47	10.45	31.1	52	Merestinib: 80 mg po qd
(II)	MER+GP	102			6.97	14.03	19.6	48	Ramucirumab: 8 mg/kg d1, 8
	GP	101			6.64	13.04	32.7	33	GP: Gemcitabine: 1000 mg/m² + Cisplatin: 25 mg/m² d1, 8
Ramaswamy et al., (26)	GP	141	53	52		8.02	44	12	GP: Gemcitabine: 1000mg /m² d1, 8+Cisplatin: 25mg/m² d1, 8
(India)	GEMOX	154	53	52		7.79	56	4	GEMOX: Gemcitabine: 1000mg /m² d1+Oxaliplatin: 100mg/m² d1

### Network Meta-Analysis Results for Overall Survival and Progression-Free Survival

**Figure 2:**
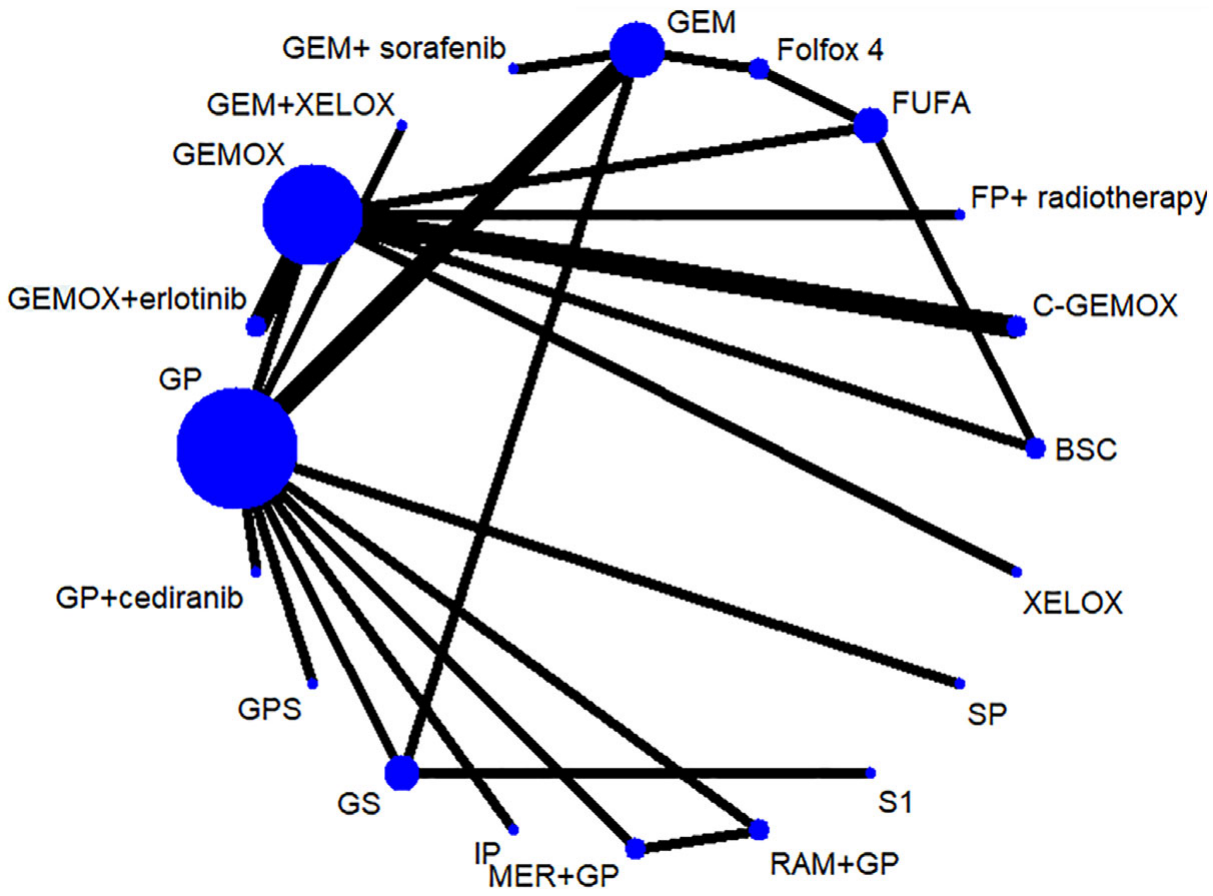
Network evidence diagram of drugs in each protocol.

**Figure 3:**
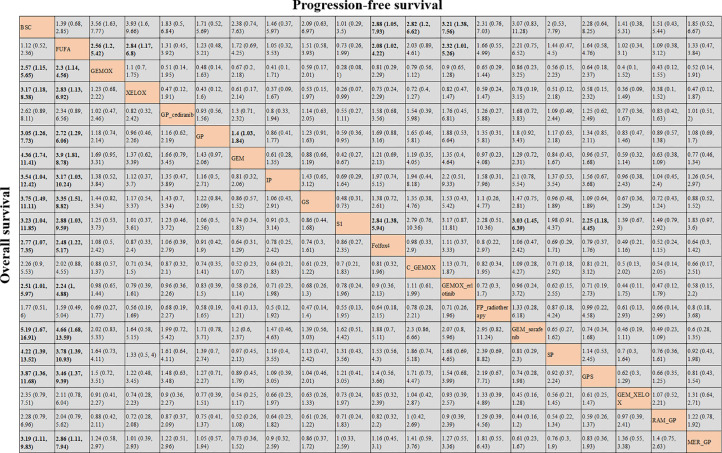
Bayesian network meta-analysis (NMA) results of overall survival (OS) and progression-free survival (PFS) of key outcome indicators for different protocols.

**Figure 4:**
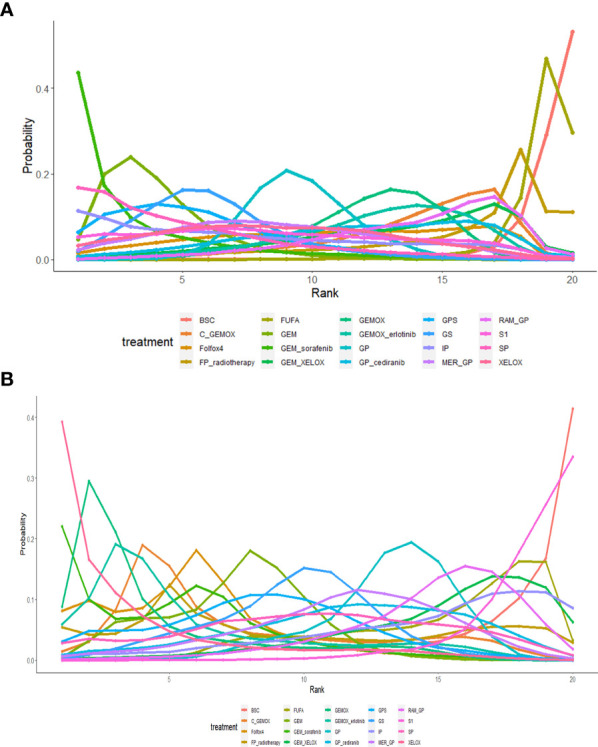
**(A)** The efficacy ranking line diagram of overall survival (OS) of each protocol; **(B)** the efficacy ranking line diagram of progression-free survival (PFS) of each protocol.

OS and PFS (3–6, 8–25, 27) were reported in the results of 23 studies involving 20 protocols; the network evidence diagrams are shown in **Figure 2**. The width of the line is proportional to the number of tests. The comparison of OS and PFS of each protocol obtained from Bayesian NMA is shown in **Figure 3**. Bold means that the results are statistically significant (p < 0.05). The results suggested that GEMOX, XELOX, GP, GEM, IP, GS, S1, Folfox4, GEMO+xerlotinib, GEM+sorafenib, SP, GPS, and MER+GP can improve patients’ OS compared with BSC or FUFA protocols. Folfox4, GEMOX+erlotinib, and C+GEMOX can improve patients’ PFS compared with BSC, which is statistically significant. There was no inconsistency between direct and indirect comparisons, and no heterogeneity was found in each protocol. Forest plots are presented in **Supplementary Figures S2A, S2B**. OS outcomes are ranked in **Figure 4A**, and the top 2 are GEM+sorafenib and GEM. PFS efficacy ranking is shown in **Figure 4B**, and the top 2 are XELOX and GEMOX protocols.

### Network Meta-Analysis Results for Objective Remission Rates

**Figure 5:**
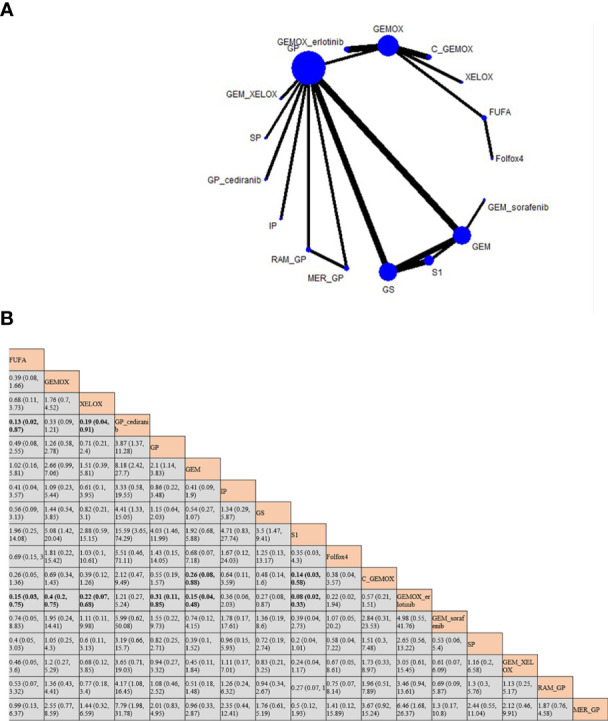
**(A)** Network evidence diagram of objective remission rate (ORR) of each protocol; **(B)** Bayesian network meta-analysis (NMA) results of ORR of each protocol.

**Figure 6:**
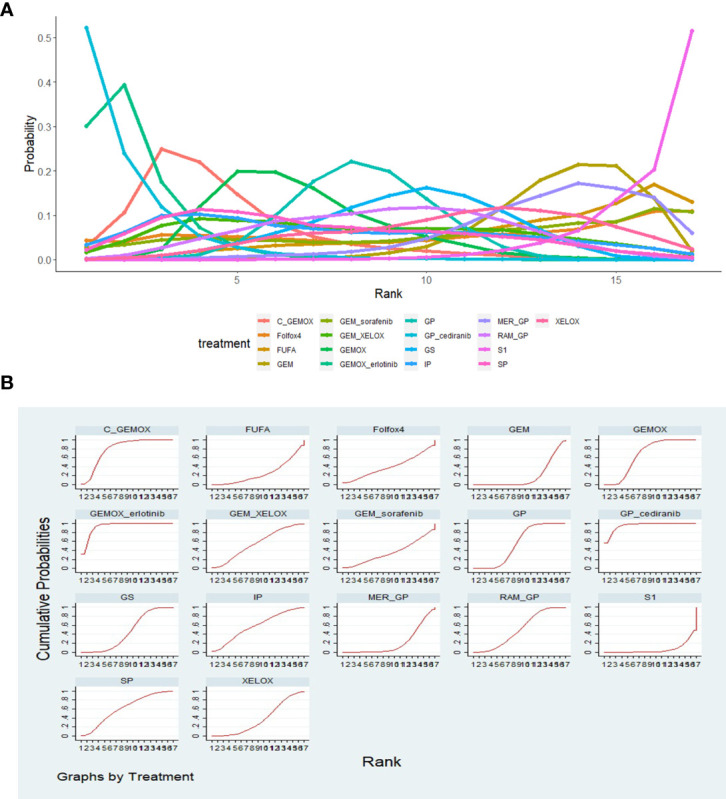
**(A)** The ranking line chart of objective remission rate (ORR); **(B)** the surface under the cumulative ranking (SUCRA) diagrams of ORR.

A total of 21 studies reported ORR (3–6, 8–18, 20, 21, 23–27) of all the studies involving 17 protocols, and the network evidence diagrams are shown in **Figure 5A**. The comparison of the efficacy of each protocol obtained from Bayesian NMA is shown in **Figure 5B**. Bold means that the results are statistically significant (p < 0.05). The results suggested that the ORR of patients with GP+cediranib protocol is higher than that of patients with FUFA, XELOX, GP, GEM, GS, S1, RAM+GP, and MER+GP protocols, and the difference is statistically significant. All included studies showed no inconsistency, heterogeneity, and bias, as shown in **Supplementary Figures S3A–S3C**. The efficacy ranking line chart and SUCRA chart of ORR are shown in **Figures 6A, B**; the top 3 are GP+cediranib, GEMOX+erlotinib, and C+GEMOX protocols (SUCRA = 95.4%, 93.8%, 79.4%, respectively).

### Network Meta-Analysis Results for Adverse Events

**Figure 7:**
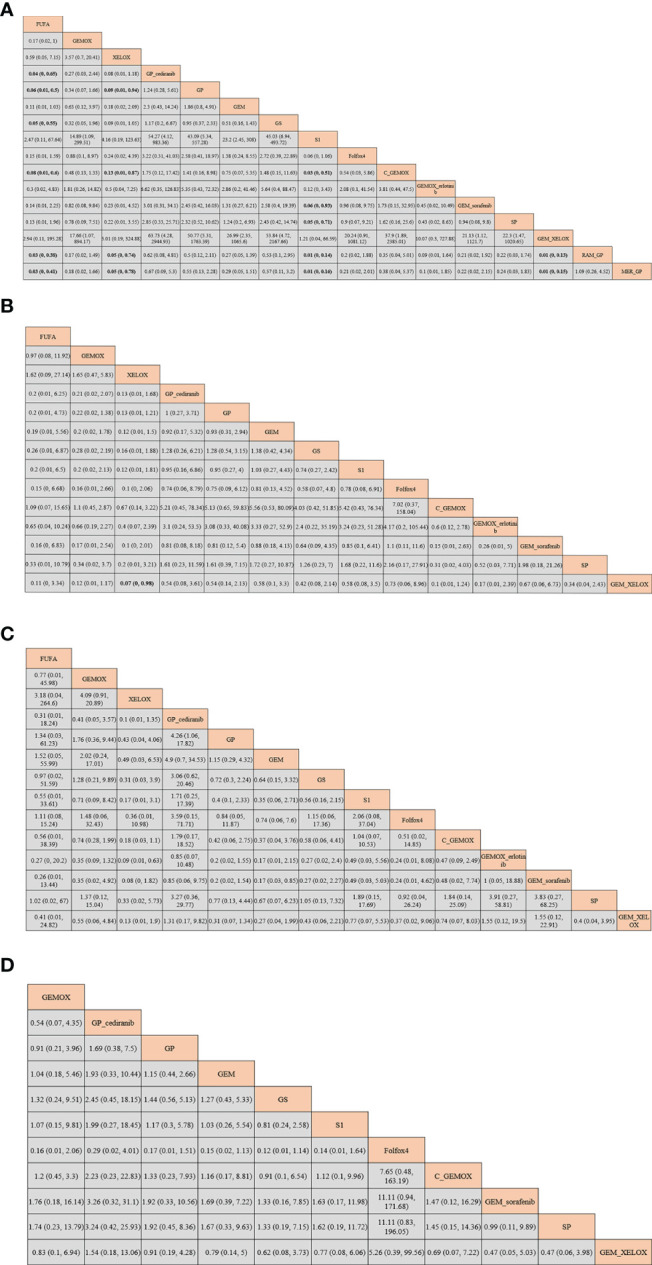
Bayesian network meta-analysis (NMA) results of adverse events (AEs) of each protocol: **(A)** neutropenia; **(B)** vomiting; **(C)** diarrhea; **(D)** fatigue.

**Figure 8:**
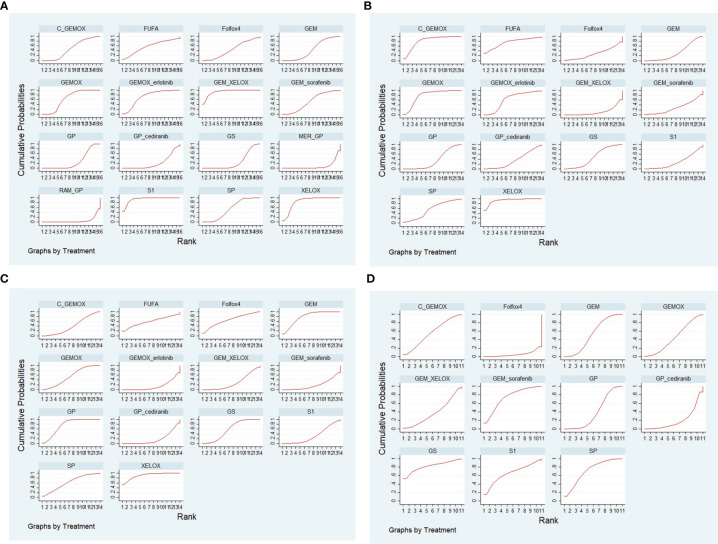
The surface under the cumulative ranking (SUCRA) diagrams of adverse events (AEs) of each protocol: **(A)** neutropenia; **(B)** vomiting; **(C)** diarrhea; **(D)** fatigue.

Based on all the studies, we selected four mostly reported AEs for subgroup analysis: neutropenia grade ≥3, vomiting, diarrhea, and fatigue. The comparison of the four AEs of each protocol obtained from Bayesian NMA is shown in **Figure 7**. We found that in neutropenia, there was reduction of neutropenia grade ≥3 when adopting GP+cediranib, GS, C+GEMOX, RAM+GP, and MER+GP than when using FUFA protocol; there was reduction of neutropenia grade ≥3 when adopting GP, C+GEMOX, RAM+GP, and MER+GP than when using XELOX protocol, and the differences are statistically significant. The probability of vomiting of XELOX is lower than that of GEM+XELOX [odds ratio (OR): 0.07; 95% CI: 0, 0.98]. There is a lower diarrhea incidence of XELOX than that of GEMOX+erlotinib (OR: 0.09; 95 CI%: 0.01, 0.63). There is a higher diarrhea incidence of GP+cediranib than that of GP (OR: 4.26; 95 CI%: 1.06, 17.82). There was no statistical difference in the remaining analysis results. The SUCRA diagram of AEs of each protocol is shown in **Figures 8A–D**. The higher the SUCRA value, the lower the incidence. The top 3 are as follows: neutropenia grade ≥3: S1, GEM+XELOX, XELOX (SUCRA: 94.3%, 93%, 84.8%, respectively); vomiting: C+GEMOX, GEMOX, FUFA (SUCRA: 81.7%, 78.8%, 74.5%, respectively); diarrhea: XELOX, GEM, Folfox4 (SUCRA: 91.2%, 78.4%, 65.1%, respectively); fatigue: GS, GEM+sorafenib, SP (SUCRA: 82.1%, 74.1%, 71.5%, respectively). All included studies showed no inconsistency, heterogeneity, and bias as shown in **Supplementary Figures S4–S7**.

### Cluster Rank Analysis of Objective Remission Rates and Adverse Events

**Figure 9:**
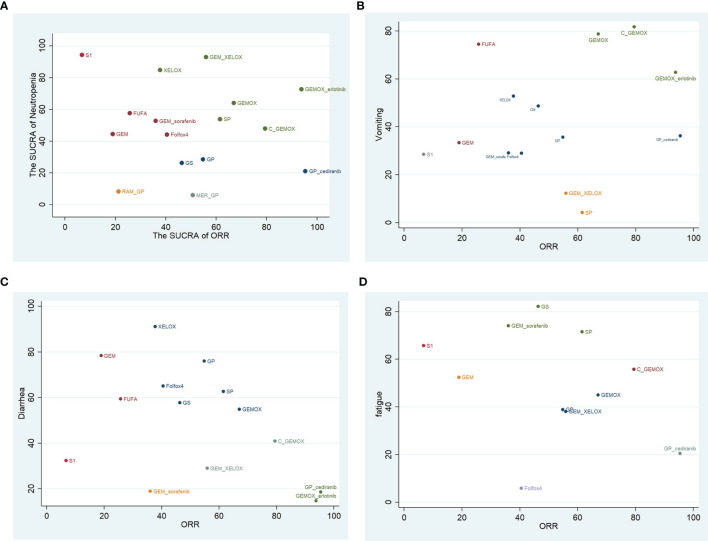
Clustered ranking plot on objective remission rate (ORR) and adverse events (AEs) both expressed as surface under the cumulative rankings (SUCRAs): **(A)** neutropenia; **(B)** vomiting; **(C)** diarrhea; **(D)** fatigue.

Based on the above NMA results, cluster rank analysis was conducted on the ORR of each intervention and the corresponding reported SUCRA of AEs (neutropenia grade ≥3, vomiting, diarrhea, and fatigue). The result demonstrated in **Figure 9** suggested that GEMOX+erlotinib owned a higher remission rate and a higher SUCRA of neutrophilic granulocytopenia and vomiting but also had a lower SUCRA of diarrhea and fatigue. Meanwhile, both GEMOX and C+GEMOX have a better remission rate and a higher AE SUCRA.

### Heterogeneity and Inconsistency Assessment

We analyze the heterogeneity and inconsistency of the results; the forest plot and funnel plot of the offset results were generated in **Supplementary Figures S2****-****S7**. Our assessment suggested minimal (I^2^ = 0%) or low heterogeneity in half of all comparisons. The high heterogeneity was detected in OS in comparisons of GS and GEMOX regimens (I^2^ = 59.8% vs. 48.4%). There was no severe heterogeneity, inconsistency analysis, and bias in the PFS, ORR, and AEs of each regimen (τ^2^ = 0 and p > 0.05).

## Discussion

This paper comprehensively compared in an NMA the efficacy and safety in advanced BTC of the most commonly used first-line BTC therapies to the best of our knowledge. The results showed that in terms of patients’ OS, the above protocols own a better efficacy to prolong patients’ OS than BSC does. There is no statistical difference between the other protocols through pairwise comparison, but it could be found that gemcitabine+sorafenib had a better effect than gemcitabine alone. XELOX and GEMOX performed better in prolonging patients’ PFS than other protocols. The three chemotherapy protocols GP +cediranib, GEMOX+erlotinib, and C+GEMOX combined with targeting can improve patients’ ORR compared with different protocols. At the same time, there were fewer occurrences of AEs in GEMOX+erlotinib and C+GEMOX. As a result, chemotherapy combined with targeting has better efficacy and lower AE incidence than chemotherapy alone. Critical headway has been made in targeted therapy acting on single or multiple targets in the treatment of advanced BTC.

KRAS mutations and epidermal growth factor receptor (EGFR) overexpression have been reported in many investigations studying advanced BTC (28, 29). Studies have shown that overexpressed EGFR (30) can be found in 38%–100% tumor samples. Cetuximab is a recombinant human-mouse chimeric IgG1 EGFR monoclonal antibody with high affinity and has been approved for treatment in patients with metastatic colorectal cancer (MCC) and head and neck cancer (31, 32). Several studies have also shown that cetuximab has a significant effect on the treatment of advanced BTC (33–35). Also, the drugs that can treat EGFR overexpression include tyrosine kinase inhibitor (TKI) erlotinib (36). This NMA confirmed that cetuximab or erlotinib could improve the ORR of patients with advanced BTC and also owned a preferable drug tolerance, but its effect on the prolongation of OS and PFS was not noticeable, which was confirmed in this meta–analysis (37). Additionally, several studies have shown that the status of KRAS genes does not seem to affect clinical results (18).

Meanwhile, we also found that GEMOX+erlotinib was better than the protocol fluorouracil+folic acid in OS, PFS, and ORR. Both previous NMAs also suggested that protocol gemcitabine was superior to protocol fluorouracil (38, 39). Cediranib combination chemotherapy in this NMA also has preferable ORR and tolerance. Cediranib is a kind of vascular endothelial growth factor receptor (VEGFR) inhibitor, which can improve the ORR of patients with advanced BTC. This has been confirmed in ABC-03 studies, but its effect on the prolongation of patients’ PFS was not pronounced (10). A meta-analysis showed that targeted drugs of EGFR are superior to those of VEGFR in prolonging patients’ PFS (37, 40).

Notably, according to the SUCRA, we found that sorafenib appeared to perform better in improving patients’ OS. When analyzing the AIO study (17), we found that 30.6% of the patients with sorafenib had hand–foot syndrome. Studies have confirmed that among patients with liver cancer receiving oral sorafenib treatment, patients with sibling syndrome had better OS (40, 41). Therefore, sorafenib combined with chemotherapy still has excellent potential to improve survival. Although gemcitabine and platinum-based chemotherapy have been established as the treatment criteria for advanced BTC, the prognosis of these tumors is poor. The need to improve treatment efficiency is stressed. Most of the recent reports or ongoing trials have assessed the tolerance and efficacy of molecular-targeted drugs used alone or in combination with gemcitabine and platinum chemotherapy (14). However, there is still a lack of evidence-based evidence on how doctors should choose many protocols. Li et al. (39) also compared the efficacy of chemotherapy protocols in his recent NMA on advanced BTC but did not compare AEs.

By using NMA, we compare the effectiveness and security of some protocols. NMA can reach the direct and indirect comparisons in RCTs, and our RCTs account for a large proportion, thus obtaining a more complete outcome data. A previous NMA has proven no statistically significant difference in toxicity between chemotherapy combined with targeted therapy and chemotherapy alone (38). By clustering the SUCRA of ORRs and AEs of each treatment, we found that targeted therapy combined with chemotherapy is in a better position in the quadrant. There seems to be a higher ORR and fewer severe agranulocytosis. In terms of severe agranulocytosis, GEMOX+erlotinib is superior to many single-use chemotherapy regimens, and its ORR is higher, such as GS, GP, Folfox4, and so on (**Figure 9A**). The occurrence of vomiting in C+GEMOX is also fewer than that in some single-use chemotherapy regimens (**Figure 9B**). In the RCT studies, the incidence of AEs between a single chemotherapy regimen and a combined chemotherapy regimen was not statistically significant. We compared the NMA method with specific chemotherapy regimens; chemotherapy plus biological therapy seem to have better curative effects and tolerance. We infer that it may be caused by the difference in the dose of chemotherapy drugs in each regimen. Further verification was necessary, but this is a good trend. This method has been used to compare the efficacy of various antitumor medicines, which increases the evidence-based basis for guiding clinical use.

NMA also has limitations in this paper. Most of the studies included did not provide detailed information. Besides, there are inevitable errors in software calculation. Some studies have failed to offer specific pathological types, and clinical stages were unable to do subgroup analysis on the subtypes or stages of BTC. Since RCT studies on cholangiocarcinoma are the minority, there are not many available data. If one of the studies is excluded for sensitivity analysis, it may prevent the NMA from forming a closed loop, thus affecting the estimation. So the possibility of bias or heterogeneity is not ruled out. We will further improve the sensitivity analysis to test the robustness of the results when more RCTs are added in the future. At the same time, tumor therapy has entered the era of immunotherapy, which has achieved amazing efficacy in lung cancer, liver cancer, and other tumors. Since many RCTs on immunotherapy have not yet published complete results, the NMA in this paper is not included in immunotherapy-based protocols. Today, there are many studies on ICIs for the treatment of advanced BTC, such as pembrolizumab, nivolumab, and other drugs combined with targeting or chemotherapy, whose efficacy remains to be seen. We look forward to an NMA on the effectiveness and safety of ICIs for patients with advanced BTC.

By using NMA, which involves more drugs in advanced BTC first-line therapy, we hope that our study can be used as a reference for clinical treatment. Also, we hope that more research and treatment drugs can be included in the future to carry out a more detailed analysis to obtain more comprehensive results, thereby selecting the optimal treatment plan for patients.

## Conclusions

Our meta-analysis revealed that chemotherapy combined with targeted therapy has better efficacy and lower incidence of AEs than chemotherapy alone. This finding may facilitate the effective treatment of patients with cancer.

## Data Availability Statement

The original contributions presented in the study are included in the article/**Supplementary Material**. Further inquiries can be directed to the corresponding author.

## Author Contributions

YJ and JM contributed to the conceptualization. YJ and JQ contributed to the methodology. YJ and JT contributed the software. YL and NM contributed to the validation. YJ and JZ contributed to the formal analysis. ZZ and LD contributed to the data curation. YJ contributed to writing–original draft preparation. ZZ contributed to writing–review and editing. YJ and CL contributed to the visualization. All authors contributed to the article and approved the submitted version.

## Funding

This research was supported by the Guangxi Natural Science Foundation (2018GXNSFAA281126).

## Conflict of Interest

The authors declare that the research was conducted in the absence of any commercial or financial relationships that could be construed as a potential conflict of interest.

## Publisher’s Note

All claims expressed in this article are solely those of the authors and do not necessarily represent those of their affiliated organizations, or those of the publisher, the editors and the reviewers. Any product that may be evaluated in this article, or claim that may be made by its manufacturer, is not guaranteed or endorsed by the publisher.

## Supplementary Material

Supplementary Figure S1
**(A)** Risk bias diagram; **(B)** General diagram of risk bias.

Supplementary Figure S2 **(A)** Heterogeneity test for inclusion studies; **(B)** node cutting method for inconsistency test for inclusion studies.

Supplementary Figure S3 **(A)** ORR inconsistency test; **(B)** closed-loop study heterogeneity test of ORR; **(C)** ORR funnel map.

Supplementary Figure S4 **(A)** agranulocytosis inconsistency test; **(B)** closed-loop study heterogeneity test of agranulocytosis; **(C)** agranulocytosis funnel map.

Supplementary Figure S5 **(A)** vomiting inconsistency test; **(B)** closed-loop study heterogeneity test of vomiting; **(C)** vomiting funnel map.

Supplementary Figure S6 **(A)** diarrhea inconsistency test; **(B)** closed-loop study heterogeneity test of diarrhea; **(C)** diarrhea funnel map.

Supplementary Figure S7 **(A)** fatigue inconsistency test; **(B)** closed-loop study heterogeneity test of fatigue; **(C)** fatigue funnel map.
