# Supplementary figures and images for: Efficacy and Safety of First-Line Chemotherapies for Patients With Advanced Biliary Tract Carcinoma: A Systematic Review and Network Meta-Analysis
Source: Front Oncol. 2021 Sep 28;11:736113. doi: 10.3389/fonc.2021.736113 (PMC8507323; doi:10.3389/fonc.2021.736113)

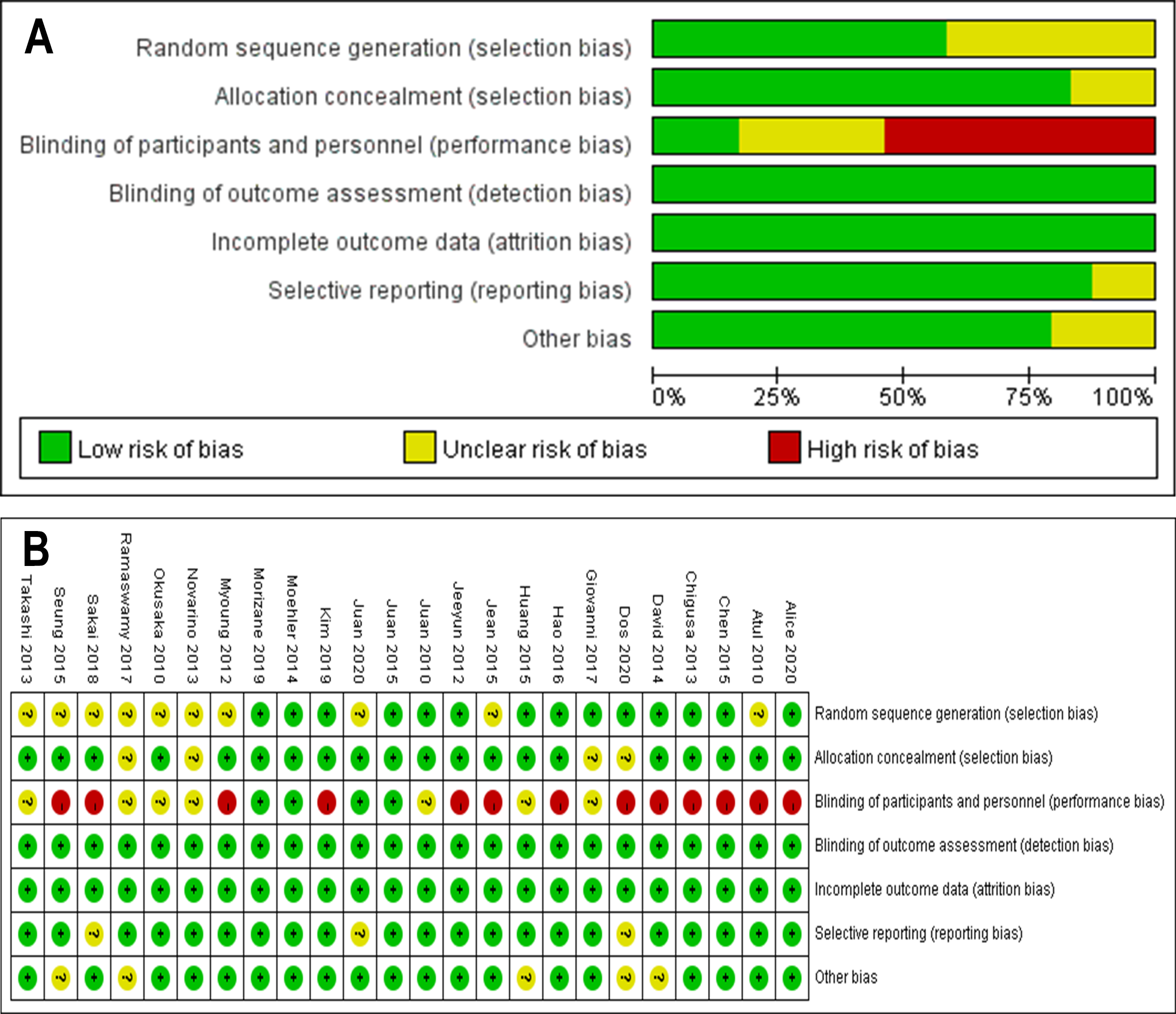

Supplement: Supplementary Figure S1 — (A) Risk bias diagram; (B) General diagram of risk bias. [file Image_1.tif]

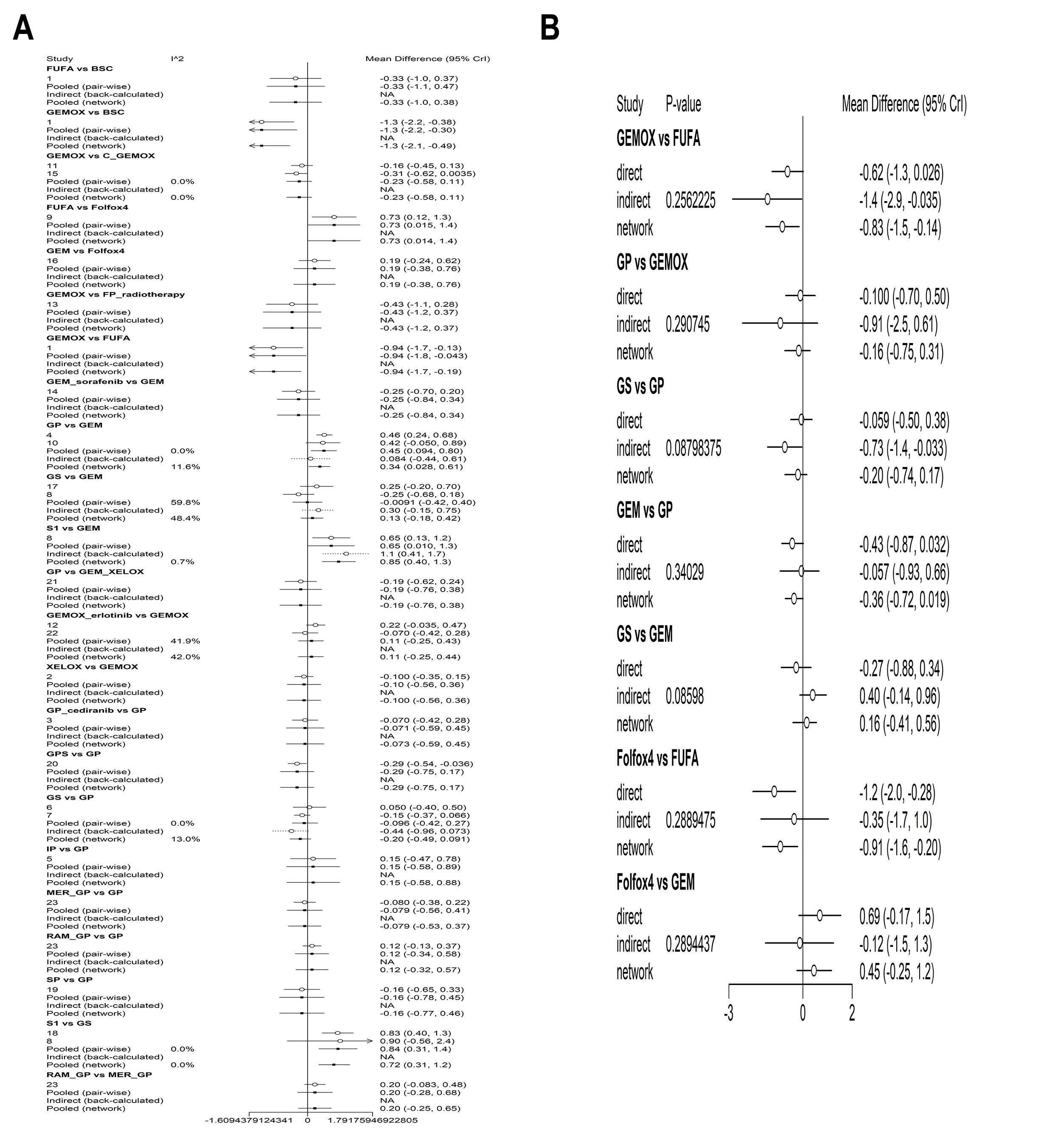

Supplement: Supplementary Figure S2 — (A) Heterogeneity test for inclusion studies; (B) node cutting method for inconsistency test for inclusion studies. [file Image_2.tif]

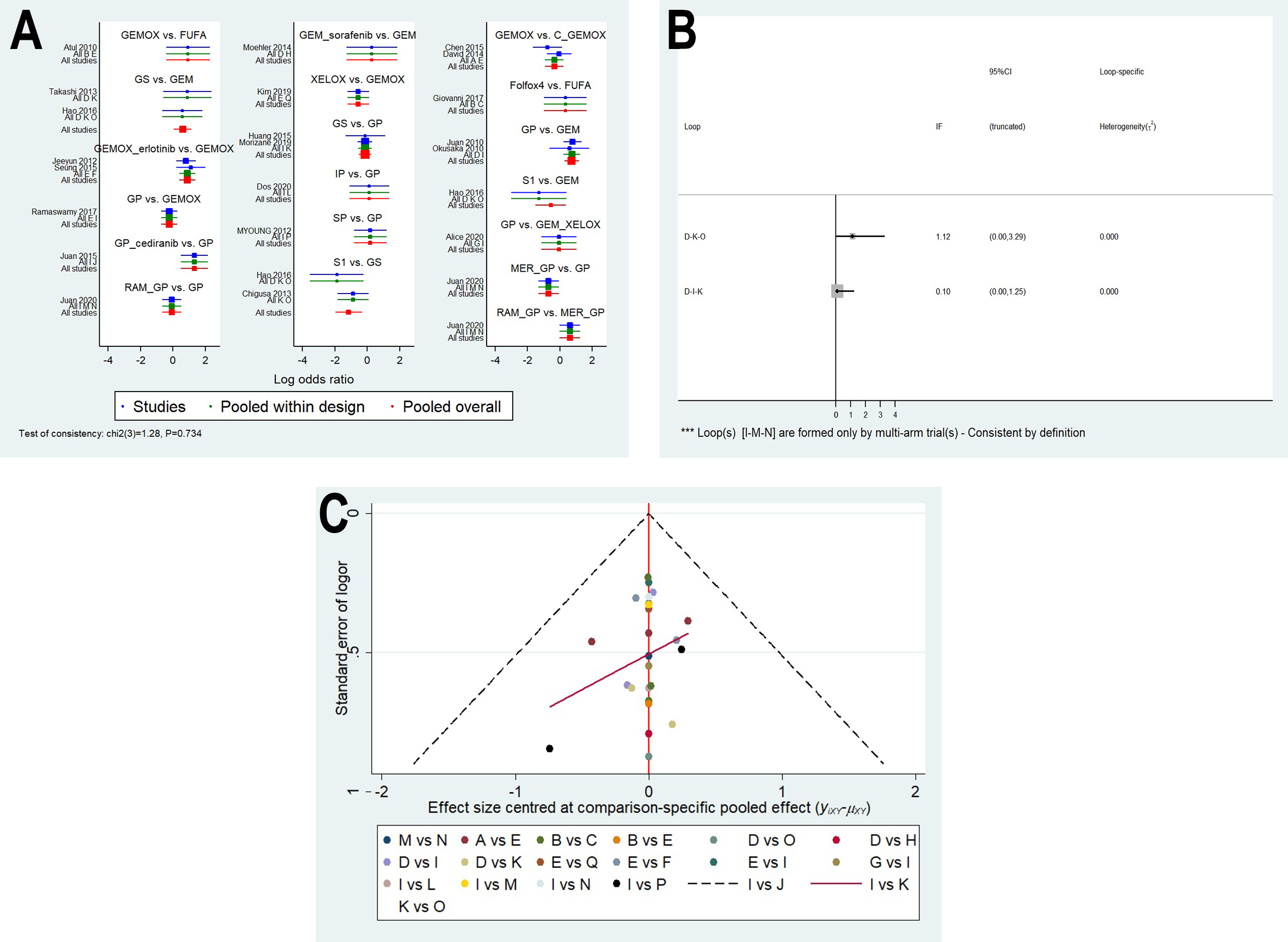

Supplement: Supplementary Figure S3 — (A) ORR inconsistency test; (B) closed-loop study heterogeneity test of ORR; (C) ORR funnel map. [file Image_3.tif]

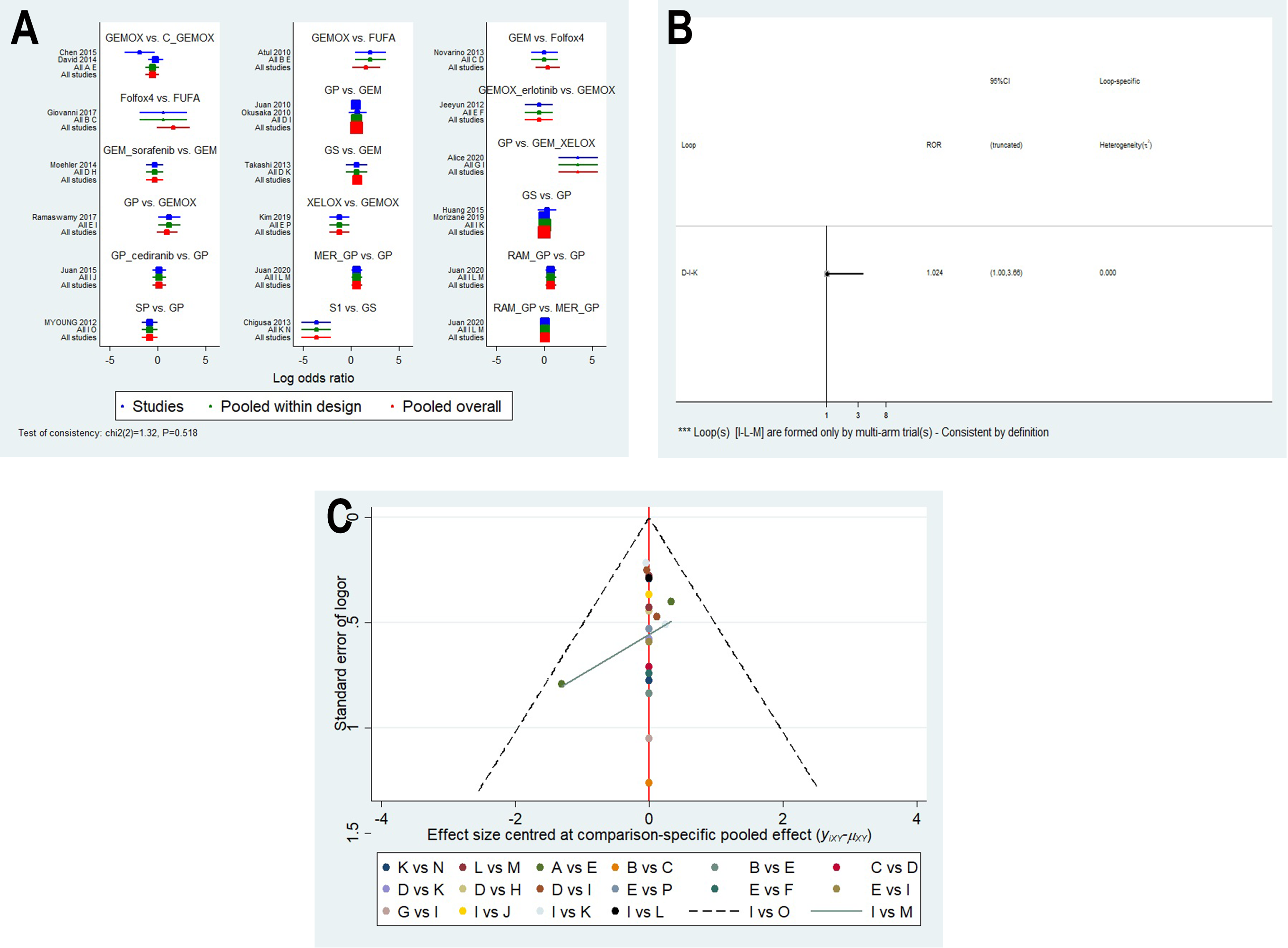

Supplement: Supplementary Figure S4 — (A) agranulocytosis inconsistency test; (B) closed-loop study heterogeneity test of agranulocytosis; (C) agranulocytosis funnel map. [file Image_4.tif]

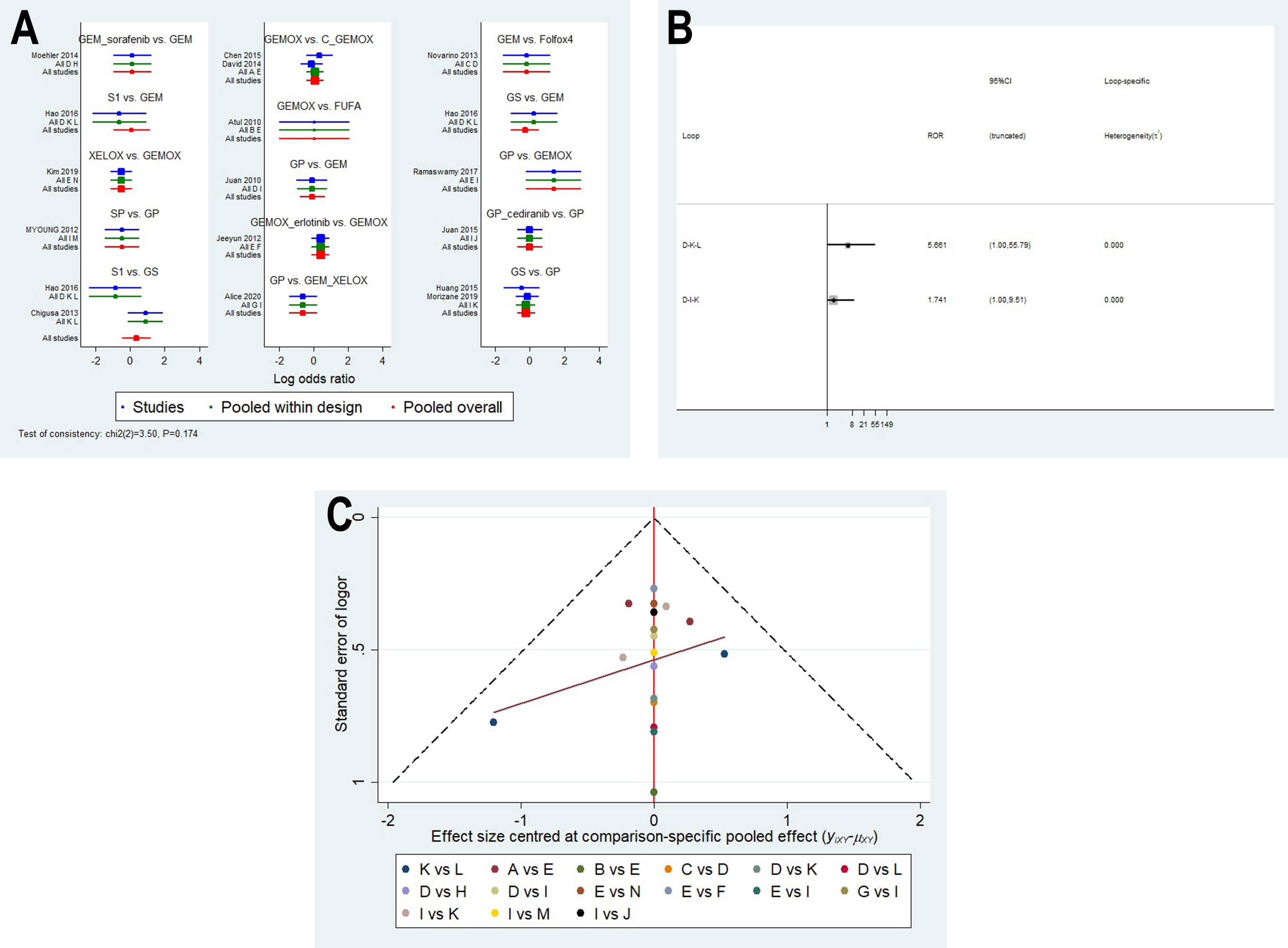

Supplement: Supplementary Figure S5 — (A) vomiting inconsistency test; (B) closed-loop study heterogeneity test of vomiting; (C) vomiting funnel map. [file Image_5.tif]

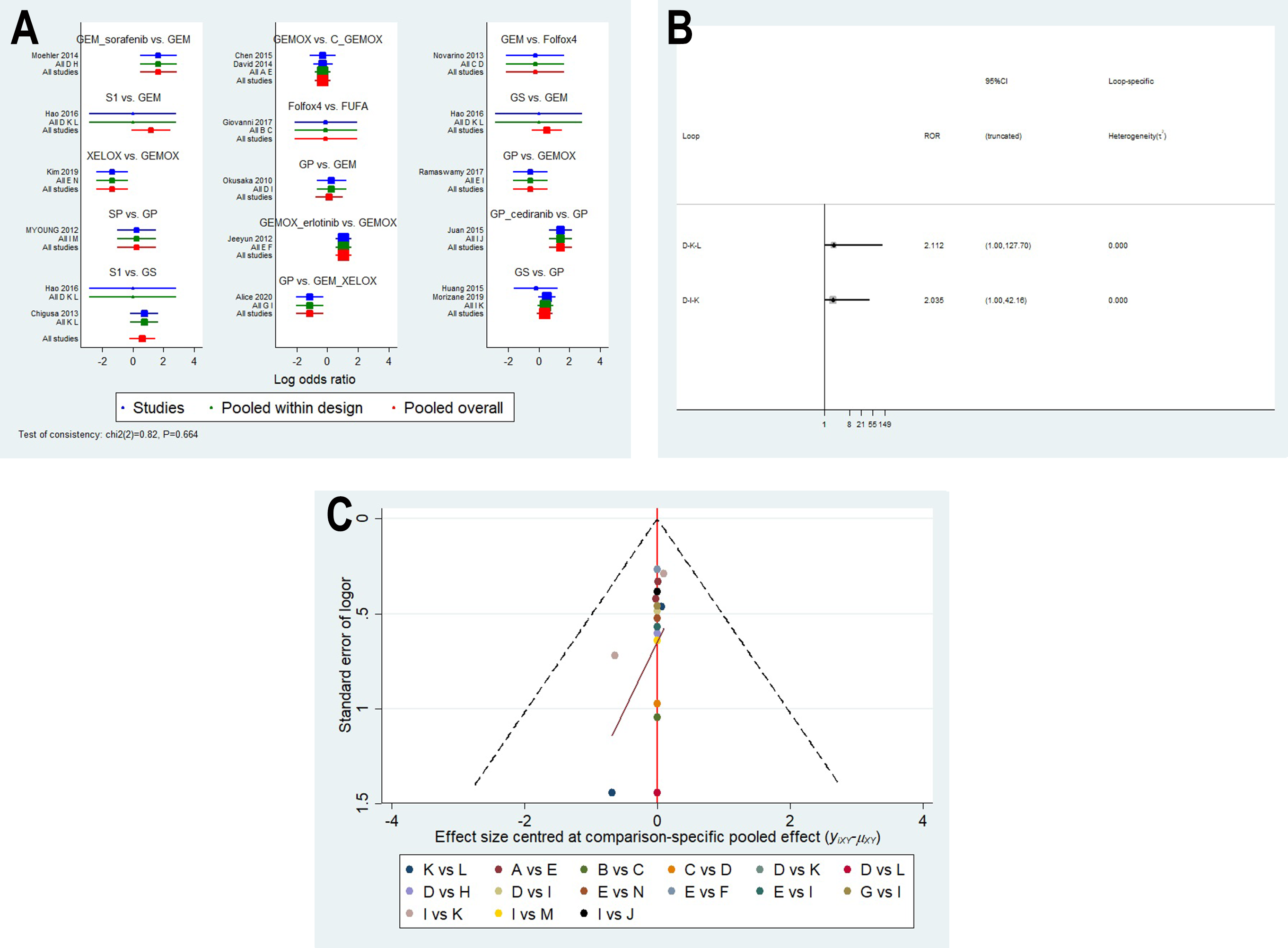

Supplement: Supplementary Figure S6 — (A) diarrhea inconsistency test; (B) closed-loop study heterogeneity test of diarrhea; (C) diarrhea funnel map. [file Image_6.tif]

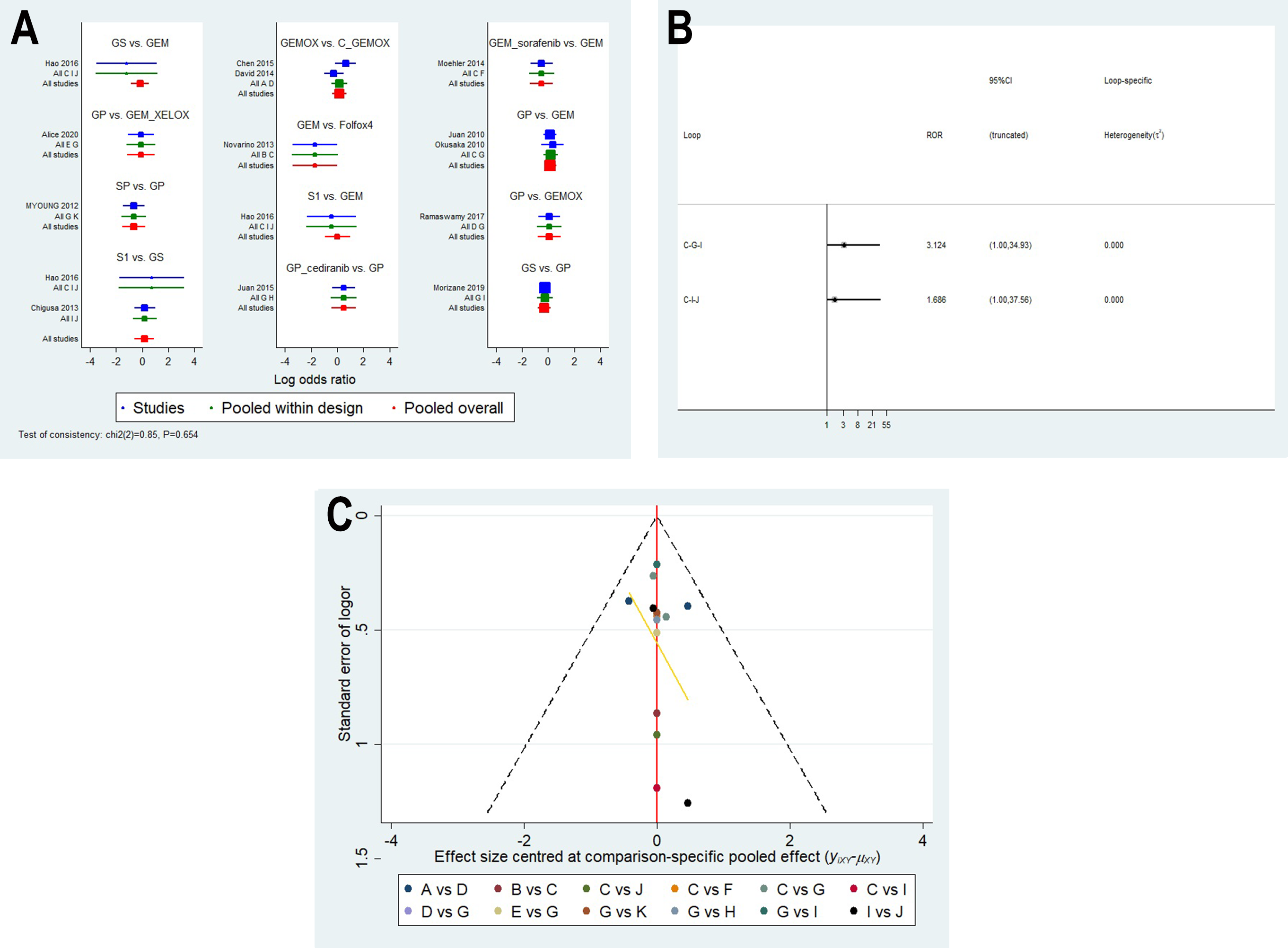

Supplement: Supplementary Figure S7 — (A) fatigue inconsistency test; (B) closed-loop study heterogeneity test of fatigue; (C) fatigue funnel map. [file Image_7.tif]
